# Impact of prenatal exposure to benzodiazepines and z-hypnotics on behavioral problems at 5 years of age: A study from the Norwegian Mother and Child Cohort Study

**DOI:** 10.1371/journal.pone.0217830

**Published:** 2019-06-06

**Authors:** Lene Maria Sundbakk, Mollie Wood, Jon Michael Gran, Hedvig Nordeng

**Affiliations:** 1 PharmacoEpidemiology and Drug Safety Research Group, Department of Pharmacy, and PharmaTox Strategic Initiative, Faculty of Mathematics and Natural Sciences, University of Oslo, Oslo, Norway; 2 Department of Epidemiology, Harvard T.H. Chan School of Public Health, Boston, MA, United States of America; 3 Oslo Centre for Biostatistics and Epidemiology, Department of Biostatistics, University of Oslo, Oslo, Norway; 4 Department of Child Development and Health, Norwegian Institute of Public Health, Oslo, Norway; Karolinska Institutet, SWEDEN

## Abstract

Many women experience anxiety or sleep disorders during pregnancy and require pharmacological treatment with benzodiazepines (BZDs) or z-hypnotics. Limited information is currently available on how prenatal exposure to these medications affects behavioral problems in children over the long term. Therefore, from a public health perspective, this issue is highly important. The present study aimed to determine whether prenatal exposure to BZDs and z-hypnotics affected externalizing and internalizing behavior problems in children at age 5 years. This study was based on The Norwegian Mother and Child Cohort Study and The Medical Birth Registry of Norway. The final study population included data for 36 401 children, from questionnaires completed by the mothers throughout the 5-year follow up. Children’s behaviors were measured at age 5, based on parental responses to The Child Behavior Checklist. Children T-scores of 63 or above were considered to indicate clinically relevant behavior problems. We applied inverse probability of treatment weighting (IPTW) and log-binomial regression models to estimate risk ratios (RRs) and bootstrapped 95% confidence intervals (CIs) with censoring weights to account for loss during follow-up. Several sensitivity analyses were performed to assess the robustness of the main results. The final sample included 273 (0.75%) children that were exposed to BZDs and/or z-hypnotics during pregnancy. The main, IPTW and censoring weighted analyses showed that prenatal exposure to BZD and/or z-hypnotics increased the risks of internalizing behavioral problems (RR: 1.35, 95% CI: 0.73–2.49) and externalizing behavioral problems (RR: 1.51, 95% CI: 0.86–2.64). However, based on sensitivity analyses, we concluded that the risks of displaying externalizing and internalizing problems at 5 years of age did not significantly increase after prenatal exposure to BZDs and/or z-hypnotics. Instead, the sensitivity analyses suggested that residual confounding and selection bias might explain the increased risks observed in the main analyses.

## Introduction

Up to 15% of women experience anxiety during pregnancy [1], and of these, 10%-26% require pharmacological treatment with benzodiazepines (BZDs) [2–4]. BZDs, like oxazepam and diazepam, are drugs prescribed for treating mental diseases, anxiety disorders, and/or sleep problems, due to their anxiolytic and sedative effects [5]. The z-hypnotics, zolpidem and zopiclone, are BZD-related drugs that are mainly prescribed as mild sedatives [6]. Both BZDs and z-hypnotics modulate the *γ*-amino butyric acid (GABA_A_) receptor [7]. When taken during pregnancy, these medications cross readily the placenta and the blood brain barrier. They act by facilitating the opening of GABA-activated chloride channels [7] present in the cortical anlage from embryonic age [8] (i.e. before week 9 after conception), and thus increasing the response to GABA. In the mature central nervous system, GABA is a neurotransmitter that acts in an inhibitory manner. At the early developmental stage, however, GABA acts in an excitatory manner and is involved in neurogenesis, including proliferation, migration, differentiation of neurons, as well as the timing of critical periods and potentially primes the earliest neuronal networks [8]. Consequently, it is biological plausible that BZDs and z-hypnotics could affect fetal neurodevelopment [9, 10].

Previous studies have investigated the neurobehavioral effects of BZDs on the brain in animals [11–14]. In brief, these studies showed a range of impaired motor development and behavioral alterations. In humans, however, few studies have investigated how prenatal BZD and z-hypnotic exposure might affect long-term neurocognitive development. Some studies have been conducted on behavior outcomes in offspring after prenatal BZD exposure [15–17], but results were conflicting. One sibling-matched (n = 10) study evaluated the teratogenic and fetotoxic potential of very large doses of medazepam, taken during an attempted suicide (60–500 mg, mean = 276 mg). However, they observed no adverse effects on the behavior status of the offspring (8–12 months) [15]. In contrast, another study on children (n = 17) born to mothers that used lorazepam, oxazepam, and/or diazepam in prescribed doses throughout pregnancy, showed reduced personal-social development in the children (18 months) [16]. Finally, a retrospective study on children (n = 15) born to mothers taking BZDs during the second half of pregnancy found no effects on behavior at ages 9–10 years [17]. All of those studies had small sample sizes and no control for the indication of maternal use.

In recent years, some larger studies have used more advanced methods in pharmacoepidemiology to address concerns about confounding and bias. A cohort study compared children prenatally exposed to BZDs and z-hypnotics (n = 104) to children exposed to maternal prenatal anxiety or phobic anxiety symptoms, but without exposure to BZDs or z-hypnotics (n = 527). They reported that prenatal BZD and z-hypnotic exposure were not independently associated with aggressive behavior or oppositional defiant disorder at 6 years of age, when maternal anxiety symptoms during pregnancy were taken into account (β: 0.23, 95% confidence interval (CI): -0.30–0.76) [18]. In contrast, another study conducted a sibling comparison with data from the Norwegian Mother and Child Cohort Study (MoBa). They evaluated internalizing and externalizing problems at 1.5 years (19 297 siblings) and 3 years (13 779 siblings). That study suggested that internalizing behaviors were slightly increased at both 1.5 years (standardized β: 0.25, 95% CI: 0.01–0.49) and 3 years (standardized β: 0.26, 95% CI: 0.002–0.5) after prenatal exposure to anxiolytics [19]. Consequently, uncertainty remains about the long-term neurodevelopmental safety of BZDs and z-hypnotics during pregnancy. This information is essential for informing women that face the decision of whether to use these medications during pregnancy.

As a follow up to the Norwegian sibling-comparison study [19], we aimed to determine whether prenatal exposure to BZDs and z-hypnotics affected externalizing and internalizing behavior problems in 5-year-old children in the MoBa. Specifically, we aimed to apply appropriate statistical methods, including propensity score (PS) methods, to control for important measured confounders and to explore the role of unmeasured confounding factors.

## Materials and methods

### Study population and data collection

This study was based on data from the MoBa study [20], which is a prospective population-based cohort study conducted by the Norwegian Institute of Public Health. Participants were recruited from all over Norway from 1999–2008. The women consented to participation in 40.6% of the pregnancies. A total of 114 500 children, 95 200 mothers, and 77 300 fathers are currently included in the cohort. The present study was based on version 9 of the quality-assured data files [21]. All data are based on prospectively self-administered questionnaires. Around gestational weeks 17 and 30 (Q1 and Q3), the mothers answered questions regarding sociodemographic characteristics, maternal health, and medication use during pregnancy. In addition, one questionnaire, completed 6 months after birth, covered all the weeks of pregnancy after week 30 (Q4). The children were followed up with questionnaires completed by the mothers at 18 months, 3 years, and 5 years after birth (Q5–Q-5year). The data were linked to the Medical Birth Registry of Norway (MBRN) via personal identification numbers. The MBRN contains information on the pregnancy, delivery, postpartum complications, interventions, and medical information regarding the infant [22]. The establishment and data collection in MoBa was previously based on a license from the Norwegian Data protection agency and approval from The Regional Committee for Medical Research Ethics, and it is now based on regulations related to the Norwegian Health Registry Act. The current study was approved by The Regional Committee for Medical Research Ethics, region South East (2015/1897). Written informed consent was obtained from all the MoBa participants prior to participation.

**Fig 1:**
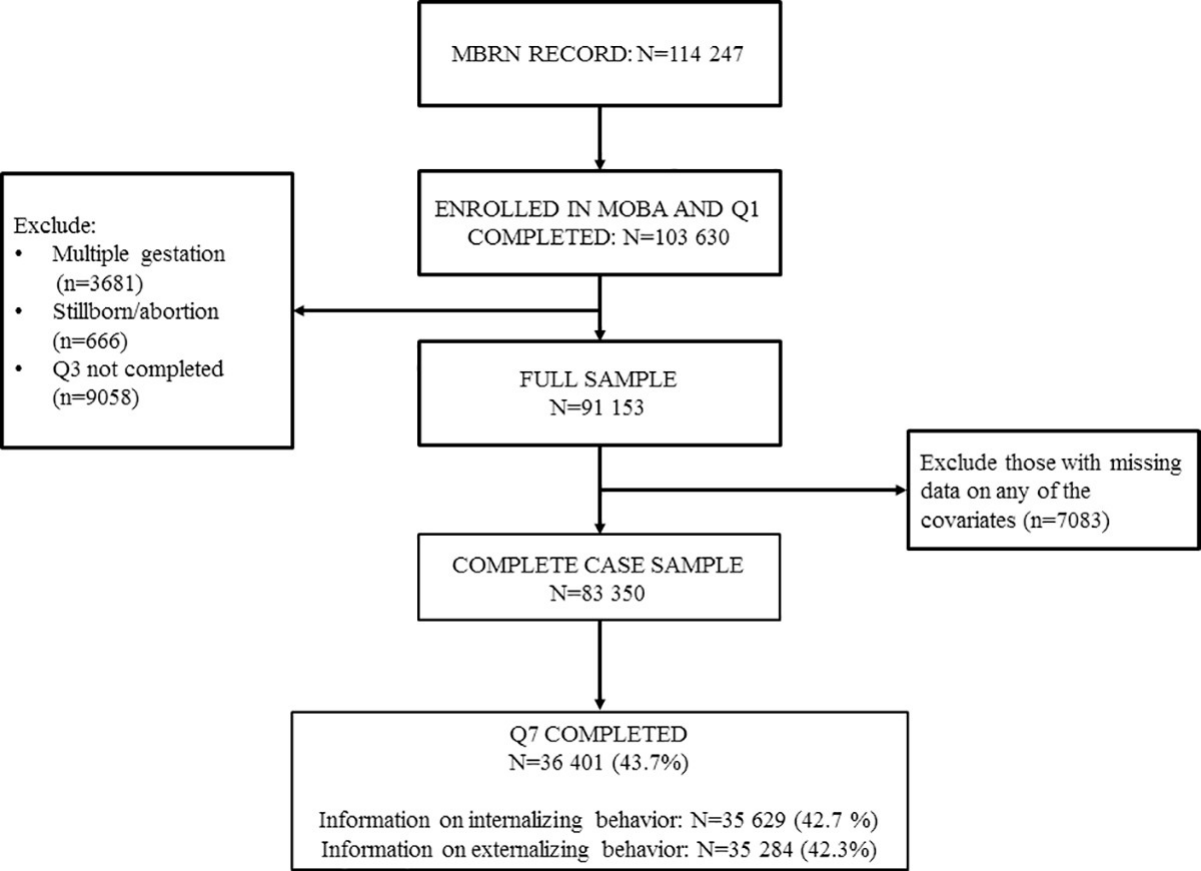
Flow chart displays the selection of study participants. MBRN, Medical Birth Registry of Norway; MOBA, Norwegian Mother and Child Cohort Study.

Our final analyses included complete case data from 36 401 live-born children, whose mothers had returned the 5-year follow up questionnaire. Fig 1 summarizes the inclusion and exclusion criteria used to select the final study population.

### BZD and z-hypnotic exposure

Information on BZD and z-hypnotic use was available from two prenatal (Q1 and Q3) and one postnatal (Q4) questionnaire. BZDs were classified according to Anatomical Therapeutic Chemical (ATC) [23] groups, including ATC groups N05BA (diazepam, oxazepam, colbazam, alprazolam), N05CD (nitrazepam, flunitrazepam, midazolam), and N03AE01 (clonazepam). Z-hypnotics included drugs within the ATC group, N05CF (zopiclone, zolpidem). In these questionnaires, women were specifically asked about a range of illnesses and health problems, including depression, anxiety, and sleeping disorders, which occurred up to 6 months prior to pregnancy and during pregnancy. For each indication, they were asked to name all the medications they used, the timing of use (week 0–4, 5–8, 9–12, 13–16, 17–20, 21–24, 25–28, and the last part of the pregnancy), and the number of days that the medication was used. Women were classified as BZD and z-hypnotic users if they reported use during pregnancy on at least one of the questionnaires.

### Externalizing and internalizing behavior

To assess child behavior at age 5 years, we used the Child Behavior Checklist, which in the MoBa is available as a shortened version of the original measure. The checklist was designed to identify problem behavior in children, and it is a validated and commonly used measure [24]. The 20 items on the shortened MoBa checklist were selected by a team of clinical and developmental psychologists, based on clinical and theoretical guidelines for externalizing and internalizing behaviors [25]. The parents reported on items that represented both internalizing behavior (consisting of the DSM_Oriented subscales: “emotionally reactive”, “anxious/depressed”, and “somatic complaints”) and externalizing behavior (consisting of the subscales: “attention problems” and “aggressive behavior”). In Q5-year, the checklist consisted of 11 items that covered externalizing problems and 9 items that covered internalizing problems. The items were rated on a three-point scale, which indicated the degree that each statement reflected the child’s behavior during the past two months: 1 = not true, 2 = somewhat or sometimes true, 3 = very true or often true. Mean scores were generated for both internalizing and externalizing behavior problems, and standardized T-scores were computed. T-scores of 63 or larger indicated that the child had clinically significant externalizing and internalizing behavior problems, according to previous recommendations [26].

### Covariates

Potential confounders were identified by reviewing the literature and constructing directed acyclic graphs [27, 28], and are presented in Table 1. Data on maternal age at delivery, parity, marital status, folate intake before and during pregnancy, child gender, birthweight, congenital malformations, and gestational age were retrieved from the MBRN. The MoBa questionnaires provided data on body mass index (BMI) before conception, smoking, illicit drug use, alcohol intake, ongoing or completed education, chronic disease, adverse life events, sleep and mental health problems, lifetime history of major depression (LTH of MD), and symptoms of depression and anxiety.

**Table 1:** Mother and child characteristics, based on whether the mother did (exposed) or did not (unexposed) use BZDs and z-hypnotics during pregnancy.

Characteristics	Study population (N = 36 401)	
*Maternal characteristics*	Exposed N = 273	Unexposed N = 36 128
Age in years, mean ± SD	31.7 ± 4.4	30.6 ± 4.4
Primiparous, n (% of N)	137 (50.2)	17 320 (47.9)
Married/cohabiting, n (% of N)	249 (91.2)	34 950 (96.7)
College/university education^a^, n (% of N)	210 (76.9)	26 414 (73.1)
Pre-pregnancy BMI, kg/m^2^; mean ± SD	23.9 ± 4.2	23.9 ± 4.1
Smoking, n (% of N)	30 (11.0)	1539 (4.3)
Alcohol intake during pregnancy^b^, n (% of N) No or minimal Low to moderate Frequent	177 (64.8) 63 (23.1) 33 (12.1)	28 130 (77.8) 5445 (15.1) 2553 (7.1)
Illicit drug use^c^, n (% of N)	10 (3.7)	189 (0.5)
Folic acid supplementation^d^, n (% of N)	176 (64.5)	23 910 (66.2)
Chronic disease^e^, n (% of N)	58 (21.2)	3680 (10.2)
LTH of MD, n (% of N)	52 (19.0)	2147 (5.9)
SCL-5^f^, mean ± SD	0.8 ± 1.5	-0.05 ± 0.8
Sleep problems, n (% of N)	127 (46.5)	5770 (16.0)
Mental health problems, n (% of N)	133 (48.7)	3787 (10.5)
Adverse life event, n (% of N) No At least one, not painful At least one, painful/very painful	66 (24.2) 51 (18.7) 156 (57.1)	15 010 (41.6) 8865 (24.5) 12 253 (33.9)
Co-medications during pregnancy, n (% of N) NSAIDs Opioids Paracetamol Antidepressants Antipsychotics Antiepileptics Triptans	40 (14.7) 35 (12.8) 187 (68.5) 57 (20.9) 19 (7.0) 7 (2.6) 11 (4.0)	2231 (6.2) 660 (1.8) 17 016 (47.1) 326 (0.9) 276 (0.8) 117 (0.3) 373 (1.0)
***Child characteristics***		
Boy, n (% of N)	141 (51.6)	18 458 (51.1)
Congenital malformation^g^, n (% of N)	12 (4.4)	1763 (4.9)
Preterm (<37 weeks)^g^, n (% of N)	16 (5.9)	1566 (4.3)
Missing	2 (0.7)	157 (0.4)
Low birth weight (<2500g)^g^, n (% of N)	15 (5.5)	872 (2.4)
Missing	1 (0.4)	20 (0.06)

SD, standard deviation; BMI, body mass index; NSAIDs, nonsteroidal anti-inflammatory drugs; SCL-5, the Hopkins Symptoms Checklist-5; LTH of MD, Life Time History of Major Depression.

^a^Highest level of either completed or ongoing education.

^b^No or minimal alcohol intake (less than once per month); Low to moderate alcohol intake (once per month to once per week); Frequent alcohol intake (more than once per week).

^c^Illicit drug use during pregnancy or the last month before pregnancy; illicit drugs included hash (exposed; unexposed: 3.7%; 0.5%), amphetamine (1.1%; 0.08%), ecstasy (1.1%; 0.02%), cocaine (1.8%; 0.07%), or heroin (0; 0.02%).

^d^Folic acid supplementation in the four weeks before pregnancy or up to week 12 of -pregnancy.

^e^Chronic diseases included asthma, diabetes treated with insulin, Crohn’s disease, arthritis, lupus, epilepsy, multiple sclerosis, and cancer.

^f^Presence of depressive or anxiety symptoms indicated on the 5-item short version of the Hopkins Symptoms Checklist (SCL-5) at gestational week 17 and/or 30.

^g^Not included in the analysis.

Maternal symptoms of depression and anxiety during pregnancy were assessed with a validated short version of the Hopkins Symptom Checklist (SCL-5) [29] at gestational weeks 17 and 30. Standardized z-scores were computed at each time point, and the average SCL-5 score was used in the analyses. The mother’s LTH of MD was reported according to five key depressive symptoms, which corresponded closely to the DSM-III criteria for lifetime major depression [30]. Additionally, women reported previous/current illnesses and health problems on the MoBa Q1, Q3, and Q4 questionnaires, which included depression, anxiety, mental health problems, and other psychological problems (hereafter, mental health problems). In addition, a number of concomitant medications were reported in Q1, Q3 and Q4: nonsteroidal anti-inflammatory drugs (NSAIDs; ATC code M01A), opioids (N02A), paracetamol (N02BE01), antidepressants (N06A), antipsychotics (N05A), antiepileptics (N03A), and triptans (N02C).

### Statistical analyses

First, we determined the baseline characteristics of the women in the final cohort, stratified by BZD and/or z-hypnotic use during pregnancy. Next, we used PSs to remove bias from measured confounders in the estimates of how BZD and/or z-hypnotic exposure affected behavioral problems in 5-year old children [31, 32]. These biases arose from systematic differences in baseline characteristics between women that did and did not use BZDs and/or z-hypnotics during pregnancy. We aimed to estimate the population average treatment effect; thus, we decided to apply stabilized inverse probability of treatment weighting (IPTW) [33]. The PSs was calculated with a logistic regression model that estimated the probability of using BZDs and/or z-hypnotics during pregnancy [33], conditional on baseline characteristics (age, marital status, parity, education, pre-pregnancy BMI, smoking, alcohol intake, folate intake, illicit drug use, chronic disease, LTH of MD, mean SCL5-score, sleeping problems, mental health problems, concomitant medication use, adverse life events) and risk factors for the outcomes (child sex). In addition, we derived the stabilized inverse probability of censoring weights (IPCW) [34], which included the same variables that we used in the IPTW model. The IPCW accounted for loss to follow-up between baseline and the 5-year assessment, and it reduced the selection bias. Both weights were estimated in the full eligible baseline sample. The final weights were the product of the IPTW and IPCW. To assess the balance of baseline covariates between exposed and unexposed groups in the sample weighted with the combined weights, we calculated the standardized weighted mean and proportion differences [35]. A difference less than 0.1 was considered a negligible difference, as previously recommended [33]. Confounders that remained unbalanced after weighting were included as covariates in the outcome model. Log-binomial regression models were fitted after applying the final weights estimate risk ratios (RRs), with a bootstrapped standard error estimation (1000 replications); this analysis was performed with the R package, survey [36–38].

### Sensitivity analyses

We performed several sensitivity analyses to assess the robustness of our primary findings. To address potential confounding by indication, the main analysis was repeated in children of mothers with mental health problems or sleep problems (N = 8475) [39].

Additionally, we restricted the analysis to include only children of women that used BZDs and/or z-hypnotics, either during pregnancy or prior to pregnancy only (N = 366). The mothers in these two groups were likely to display similar mental health conditions; therefore, we assumed that this restriction would contribute to disentangling the effects of the underlying maternal conditions from the potential effects of the medications.

We also performed a negative control exposure analysis to detect residual confounding [40]. We compared children of mothers that used BZDs or z-hypnotics before pregnancy, but not during pregnancy, to children of mothers that did not use BZDs or z-hypnotics before or during pregnancy. The time before pregnancy was not considered an etiologically relevant exposure period; thus, any differences between groups in this analysis would likely be due to residual confounding [40]. Separate IPTW models were fitted to data for each of the groups in the sensitivity analyses [41].

The data in the 5-year sample might have been subject to selection bias, because some participants were lost to follow-up after 3 years. It was possible that only children with more serious behavioral problems were lost to follow up between 3 and 5 years; in that case, we expected a bias towards the null. To estimate the potential impact of the loss to follow-up on externalizing behavior problems, we performed a probabilistic bias analysis [42]. We calculated the proportions of children with and without externalizing behaviors problems at 3 years that remained in the study at the 5-year follow-up. Based on simple bias analyses, with the selection proportions estimated from the 3-year sample and hypothesized selection proportions, we assigned a trapezoidal probability distribution of the selection odds ratio (OR) with 10 000 simulations (min OR: 0.74, mode 1 OR: 1.02, mode 2 OR: 1.25, max OR: 1.59). The scenarios explored are presented in detail in S2 Appendix.

In addition, we repeated the analysis after excluding individuals from regions of the propensity score distribution with no overlap. This procedure ensured fulfilment of the positivity assumption [31, 33]. Lastly, we applied a 1:4 nearest neighbor propensity score matching to estimate the average effect of the medication on behavior in the population of children that were exposed during pregnancy [33].

All analyses were performed with R version 3.4.4 [43].

## Results

Our primary study population consisted of 36 401 pregnancies. Of these, 273 (0.75%) children were exposed to BZDs and/or z-hypnotics during gestation. S1 Table presents an overview of the number of individuals that used BZDs and/or z-hypnotics, and those that used different compounds, in different time windows. The most common type of medication used during pregnancy was a BZD-anxiolytic (n = 140), specifically oxazepam (n = 73) and diazepam (n = 69); the next most common type was a z-hypnotic (n = 131), specifically zopiclone (n = 113). Most women used BZDs and z-hypnotics for mental health (46.9% (n = 128)) and/or sleeping problems (21.2% (n = 58)) (S2 Table).

There were some important baseline differences between women that did and did not use BZDs or z-hypnotics during pregnancy (Table 1). Compared to women that did not use BZDs or z-hypnotics, those that did use these drugs were somewhat older and were more likely to smoke, use alcohol, and use illicit drugs. In addition, the latter group used more concomitant medications, including NSAIDs, opioids, antidepressants, and antipsychotics, and they had a higher prevalence of selected health conditions, including symptoms of anxiety and depression.

### Internalizing behavior problems

**Fig 2:**
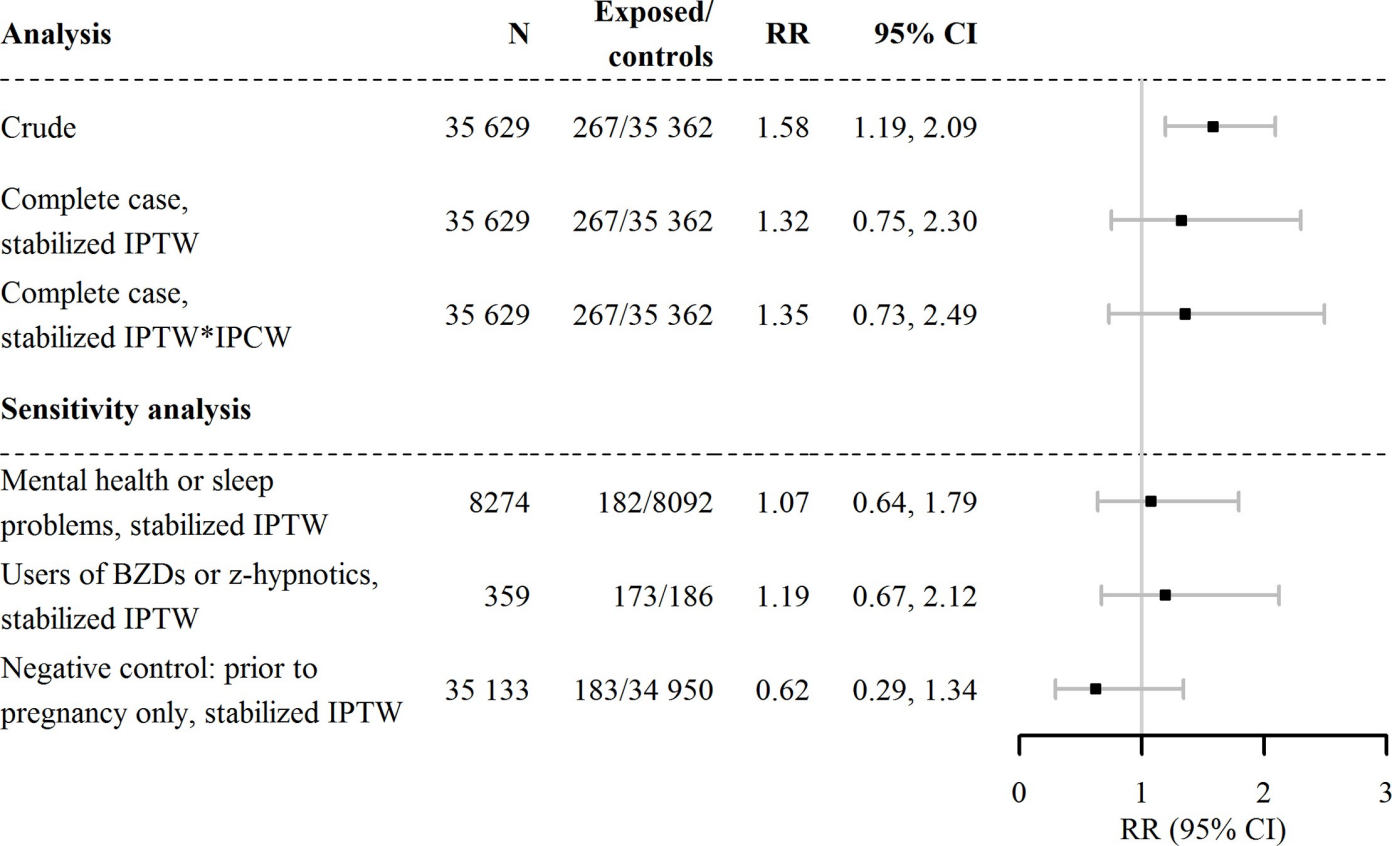
Estimated risk that prenatal exposure to BZDs and z-hypnotics could increase the probability that a child will display internalizing behavior. RR, risk ratio; CI, confidence interval; IPTW, inverse probability of treatment weights; IPCW, inverse probability of censoring weights; BZDs, benzodiazepines.

The study included 35 629 children with complete information on internalizing behavior at 5 years of age. Of these, 267 (0.75%) children were exposed to BZDs and/or z-hypnotics during gestation. Of these, 44 (16.5%) children displayed internalizing behavior problems at 5 years. In contrast, among the children that were not prenatally exposed to BZDs or z-hypnotics, 3692 (10.4%) displayed internalizing behavior problems at 5 years. In the crude analysis, BZD and/or z-hypnotic exposure was associated with an increased risk of internalizing behavior (RR: 1.58, 95% CI: 1.19–2.09), but after adjusting for potential confounders, through IPTW and censoring weighting, the increase in risk associated with BZDs and/or z-hypnotics was attenuated (RR: 1.35, 95% CI: 0.73–2.49; Fig 2).

### Externalizing behavior problems

**Fig 3:**
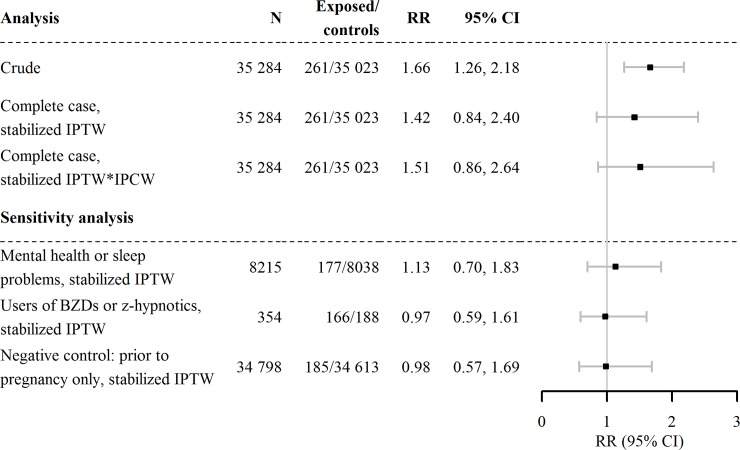
Estimated risk that prenatal exposure to BZDs and z-hypnotics could increase the probability that the child will display externalizing behavior. RR, risk ratio; CI, confidence interval; IPTW, inverse probability of treatment weights; IPCW, inverse probability of censoring weights; BZDs, benzodiazepines.

We included 35 284 children with complete information on externalizing behavior at 5 years of age. Of these, 261 (0.74%) children were prenatally exposed to BZDs and/or z-hypnotic. Of these, 43 (16.5%) children displayed externalizing behavior problems at 5 years. In contrast, among the children that were not exposed to BZDs or z-hypnotics during gestation, 3484 (9.9%) displayed externalizing behavior problems at 5 years. As shown in Fig 3, we observed an increased risk of externalizing behavior associated with prenatal exposure to BZDs and/or z-hypnotics (RR: 1.66, 95% CI: 1.26–2.18) in the crude analysis. Furthermore, after adjustment through IPTW and censoring weighting, the increase in risk associated with BZDs and/or z-hypnotics was attenuated (RR: 1.51, 95% CI: 0.86–2.64).

### Sensitivity analyses

We estimated the risks associated with different factors in the sensitivity analyses. These analyses produced different results from those obtained in the main analysis (Figs 2 and 3). First, we analyzed only the group of children with mothers that had mental health and/or sleep problems. We found lower risks compared to the main analysis (externalizing: RR: 1.13, 95% CI: 0.70, 1.83, internalizing: RR: 1.07, 95% CI: 0.64, 1.79). Then, we compared women that used BZDs and/or z-hypnotics during pregnancy to those that discontinued BZDs and/or z-hypnotics before pregnancy. We found no difference in the child’s externalizing behavior problems at 5 years of age (RR: 0.97, 95% CI: 0.59, 1.61). The same comparison showed an attenuated risk estimate for the child’s internalizing behavior compared to the main analysis (RR: 1.19, 95% CI: 0.67, 2.12). Moreover, the negative control analysis showed no increased risk of internalizing or externalizing behavior problems associated with mother’s use of BZDs and/or z-hypnotics before pregnancy, as expected. Finally, the probabilistic analysis resulted in a corrected OR of 1.56, 95% CI: 1.20–2.15 (conventional OR: 1.79, 95% CI: 1.29–2.48).

When we analyzed only individuals in the overlapping regions of the propensity score distribution, we found effect estimates almost identical to those found in the main analyses. Moreover, the effect estimates within the matched samples were similar to the results of the IPTW and censoring weighted analyses.

## Discussion

In this large prospective follow-up study of 36 401 pregnancies, we observed a modestly increased risk of internalizing and externalizing behavior problems in 5-year-old children born to mothers that used BZDs and/or z-hypnotics during pregnancy. The effect size was somewhat larger for externalizing problems than for internalizing problems. In the IPTW and censoring weighted analyses of both internalizing and externalizing problems, the confidence intervals were wide and included 1. Moreover, the larger portions of the intervals were above 1, which might be of concern; however, our sensitivity analyses suggested that residual confounding and/or selection bias might have explained some of our results.

To the best of our knowledge, only three previous studies have addressed long-term behavioral outcomes in children after prenatal exposure to BZDs and/or z-hypnotics. Our findings should be interpreted in light of findings from a previous study that showed an increased risk of internalizing problems at 3 years of age [19]. Moreover, that study revealed a small increased risk of internalizing problems at both 1.5 years and 3 years of age associated with prenatal BZD-anxiolytics exposure. In contrast, they found that z-hypnotic exposure was not associated with either externalizing or internalizing problems. However, those authors interpreted their results with caution; they stated that residual, unmeasured confounding could not be ruled out. Jointly, those previous results and our present results are clinically important results, because they might provide a basis for clinicians and women in making evidence-based decisions about the use of these medications during pregnancy.

Our findings were consistent with previous findings in studies that included school-age children. One study demonstrated that children exposed prenatally to BZDs and/or z-hypnotics had higher scores of oppositional defiant disorder and aggressive behavoir at 6 years of age. However, those associations were explained by maternal anxiety symptoms during pregnancy [18]. In another study, no association was found between BZD exposure and children’s school behavior at ages 9–10 years [17].

Assuming 5 million births in the EU each year, and a prevalence of BZD use in pregnancy between 1.5% and 3% [2, 3], we estimated that approximately 100 000 children are exposed annually to BZDs during gestation in the EU. Although BZDs have been on the market since the 1960s, only three studies world-wide have assessed their long-term neurodevelopmental safety, in less than 1000 exposed children. This low number of studies is alarming, but not surprising, given the complexity of studying these medications. They are used episodically and for a range of conditions with highly variable symptom severity during pregnancy. Moreover, we must rely on maternal reporting, because data from prescription fillings will not, most likely, reflect the timing that medications were used during pregnancy. Also, these medications are most commonly used concomitantly with a wide range of other psychotropic and analgesic medications, and also with recreational substances, which may also impact fetal brain development [2, 3]. All these factors make it challenging to identify individual drug effects. One Norwegian study found that of the women who were dispensed either a BZD or a z-hypnotic during pregnancy, 1 out of 5 were also dispensed an opioid concomitantly, and 1 out of 5 women were co-medicated with an antidepressant [2]. These factors add to the challenges of studying childhood behavioral disorders, which can be subtle and difficult to measure and may change as the child develops [44].

To assess the possible impact of unmeasured confounding, and particularly, confounding by indication, we carried out several sensitivity analyses. When we stratified by the indication for BZD and z-hypnotic exposure (e.g., anxiety, sleep problems), we found attenuated estimates compared to the main analysis. This subgroup analysis revealed a small increased risk of internalizing behavior problems, but no increased risk of externalizing behavior problems, associated with BZD and/or z-hypnotic exposure. This finding was consistent with previous findings [18, 19]. Taken together, our sensitivity analyses suggested that the findings in the main analysis might be explained by residual confounding, particularly confounding by underlying maternal illness.

Selection bias could have affected our results in several ways. First, the sample of women that consented to participate at baseline could have been systematically different from those present at the 5-year follow-up, particularly in terms of depression and anxiety severity. To address this issue, we used IPCW outcome models. Second, it was possible that women with children that had more severe behavioral problems might have been less likely to participate in the 5-year follow-up. We conducted a probabilistic bias analysis to assess whether this could explain our findings. In addition, selection bias due to loss to follow-up could have affected our results. Although we applied IPCW to account for this potential bias, censoring weights could only account for measured factors associated with the loss to follow-up.

The present study also had other limitations. First, the child’s behavior was reported by the parents, and reporting may vary with the severity of the mother’s mental illness. Second, even in such a large birth cohort, the sample size was not sufficiently large to perform analyses for specific trimesters, medication groups, or individual substances, or to perform sibling-analysis to account for familial confounding. Thirdly, our exposure definition relied on maternal reporting of BZDs and/or z-hypnotics use. An alternative could be to use the date of prescription or filling of a prescription, and to classify as exposed any periods covered by that prescription. This method is valid for medications used consistently over time (e.g. statins), or for acute medications taken for a specific time-limited indication (e.g. antibiotics); however, since BZDs/z-hypnotics are taken episodically and as-needed, this exposure definition is likely more often incorrect than self-report [45]. Interestingly, a previous MoBa sub-study validating maternal self-reported smoking in pregnancy to urinary cotinine measurements, show high validity of maternal reporting with a sensitivity of 82% and specificity of 99% [46]. A similar reporting could possibly be expected for psychotropic medication. Furthermore, we did not have any dose information, and consequently, we could not assess dose-response relationships. Due to low numbers, we could not perform analyses on the time-varying effect of the drug exposure. Lastly, the MoBa population might not be representative of the general population; indeed, the women enrolled in the study were known to be highly educated, healthy women [47]. Consequently, these findings must be replicated in larger studies, and in other countries, because we might not find the same results in other parts of the world.

From an epidemiological standpoint, this study demonstrated the benefits of using a dataset with detailed information. Moreover, our findings showed the importance of performing sensitivity analyses to assess the robustness of the main findings.

Taken together, the results from this study and previous studies are reassuring. Our findings suggested that externalizing and internalizing problems at 5 years of age were not significantly increased after prenatal exposure to BZDs and/or z-hypnotics. Our sensitivity analyses suggested that residual confounding and selection bias might explain the increased risks observed in the main analyses.

Recent initiatives suggest that it is important to consider a spectrum of neurodevelopment, not just diagnostic categories [44]. In pre-school age children, psychometric instruments may capture subtle effects in children that may be too young to have received a diagnosis. For example, the median age of ADHD diagnosis in the Norwegian population is around 7–9 years [48]. Moreover, a recent study using data from the MoBa demonstrated that the Child Behaviour Checklist at 5 years of age was useful for predicting a later ADHD diagnosis [49].

Establishing neurodevelopmental safety requires assessing a wide variety of outcomes important for the child’s daily function. Future studies on prenatal BZD and/or z-hypnotic exposure should focus on other neurodevelopmental outcomes, including psychomotor, cognitive, behavioural and emotional functioning, to give a more complete picture of the impact that these medications may have on human neurodevelopment. Brain development continues into early adulthood [50] and some problems will not be detectable until adolescence, when more complex tasks are required [51]. We therefore recommend that future studies should employ both psychometric instruments and clinical diagnosis to detect disorders that may have developmental origins, but may not be detected or diagnosed before school-age or adolescence. Future studies should also focus on the genetic factors that might confound an association between maternal psychotropic medication use during pregnancy and behavioral outcomes in the child.

## Supporting information

S1 Table Use of specific BZDs and z-hypnotics before and during pregnancy. (PDF)

S2 Table Primary disorders for the women who used BZDs and/or z-hypnotics before or during pregnancy. (PDF)

S3 Table Characteristics of the estimated stabilized IPTW and IPCW in samples with complete information on the child’s internalizing and/or externalizing behavior. (PDF)

S4 Table Balance between exposed and unexposed in the stabilized weighted samples with complete information on the child’s internalizing and externalizing behaviors. (PDF)

S5 Table Characteristics of mothers with mental health and/or sleep problems, and mothers that used BZD and/or z-hypnotics during pregnancy. (PDF)

S1 Appendix Additional information on “Materials and methods”. (DOCX)

S2 Appendix Bias analysis. (DOCX)

S1 Fig Bias analysis of the potential impact of selection bias due to loss to follow-up among children exposed to BZDs or z-hypnotics prenatally that exhibited externalizing behavior problems at 5 years of age. BZD, benzodiazepine; z-hyp, z-hypnotics; Ext. problems, externalizing problems; OR, odds ratio.
(TIF)

S2 Fig Bias analysis of the potential impact of selection bias due to loss to follow-up among children not exposed to BZDs or z-hypnotics prenatally that exhibited externalizing behavior problems at 5 years of age. BZD, benzodiazepine; z-hyp, z-hypnotics; Ext. problems, externalizing problems; OR, odds ratio.
(TIF)
