# Supplementary material for: Impact of prenatal exposure to benzodiazepines and z-hypnotics on behavioral problems at 5 years of age: A study from the Norwegian Mother and Child Cohort Study
Source: PLoS One. 2019 Jun 6;14(6):e0217830. doi: 10.1371/journal.pone.0217830 (PMC6553737; doi:10.1371/journal.pone.0217830)
Supplement: S1 Table — (PDF) [file pone.0217830.s001.pdf]

S1 Table. Use of specific BZDs and z-hypnotics before and during pregnancy.

	Study population (N=36 401)					
**Medication**	**Before pregnancy n (% of N)**	**Before pregnancy only, n (% of N)**	**1st trimester n (% of N)**	**2nd trimester n (% of N)**	**3rd trimester n (% of N)**	**Anytime during pregnancy n (% of N)**
Any BZD and/or z-hypnotic	289 (0.79)	191 (0.52)	162 (0.45)	113 (0.31)	87 (0.24)	273 (0.75)
**Subgroups**						
BZD-anxiolytics	150 (0.41)	113 (0.31)	70 (0.19)	57 (0.16)	45 (0.12)	140 (0.38)
Diazepam (N05BA01)	95 ()	77 ()	40 ()	27 ()	14 ()	69 ()
Oxazepam (N05BA04)	56 ()	38 ()	28 ()	31 ()	32 ()	73 ()
Clobazam (N05BA09)	2	0	2	1	0	3
Alprazolam (N05BA12)	2	1	2	0	0	2
BZD-hypnotics	21 (0.06)	15 (0.04)	11 (0.03)	3 (0.01)	1 (0.003)	13 (0.04)
Nitrazepam (N05CD02)	8	4	6	3	1	8
Flunitrazepam (N05CD03)	13	11	3	0	0	3

Midazolam (N05CD08)	0	0	2	0	0	2
BZD-antiepileptics<br><br>Clonazepam (N03AE01)	10 (0.03)	3 (0.01)	7 (0.02)	8 (0.02)	4 (0.01)	9 (0.02)
Z-hypnotics	135 (0.37)	88 (0.24)	89 (0.24)	52 (0.14)	44 (0.12)	131 (0.36)
Zopiclone (N05CF01)	111	75	74	44	37	113
Zolpidem (N05CF02)	25	13	16	9	7	21

BZD, benzodiazepine.
