# Supplementary material for: Impact of prenatal exposure to benzodiazepines and z-hypnotics on behavioral problems at 5 years of age: A study from the Norwegian Mother and Child Cohort Study
Source: PLoS One. 2019 Jun 6;14(6):e0217830. doi: 10.1371/journal.pone.0217830 (PMC6553737; doi:10.1371/journal.pone.0217830)
Supplement: S2 Table — (PDF) [file pone.0217830.s002.pdf]

S2 Table. Primary disorders for the women who used BZDs and/or z-hypnotics before or during pregnancy.

	**Mental health problems, n (% of N)**	**Sleeping problems, n (% of N)**	**Pain, n (% of N)**	**Other not specified indication, n (% of N)**
**Medication exposure any time during pregnancy**				
**Any BZD and/or z-hypnotic, N=273**	128 (46.9)	58 (21.2)	83 (30.4)	4 (1.5)
**Subgroups**				
BZD-anxiolytics, N=140	75 (53.6)	21 (15.0)	44 (31.4)	0
BZD-hypnotics, N=13	4 (30.8)	3 (23.1)	6 (46.2)	0
BZD-antiepileptics, N=9	5 (55.6)	2 (22.2)	2 (22.2)	0
Z-hypnotics, N=131	59 (45.0)	35 (26.7)	33 (25.2)	4 (3.1)
**Medication exposure before pregnancy only**				
**Any BZD and/or z-hypnotic, N=191**	95 (49.8)	27 (14.1)	65 (34.0)	4 (2.1)

BZD, benzodiazepine.
