# Supplementary material for: Impact of prenatal exposure to benzodiazepines and z-hypnotics on behavioral problems at 5 years of age: A study from the Norwegian Mother and Child Cohort Study
Source: PLoS One. 2019 Jun 6;14(6):e0217830. doi: 10.1371/journal.pone.0217830 (PMC6553737; doi:10.1371/journal.pone.0217830)
Supplement: S3 Table — (PDF) [file pone.0217830.s003.pdf]

S3 Table. Characteristics of the estimated stabilized IPTW and IPCW in samples with complete information on the child's internalizing and/or externalizing behavior.

Sample	Estimated IPTW		Estimated IPCW		Estimated IPTW*IPCW	
	Mean (SD)	Min-Max	Mean (SD)	Min-Max	Mean (SD)	Min-Max
Internalizing sample	1.00 (0.10)	0.01-7.66	1.00 (0.26)	0.64-3.37	1.00 (0.29)	0.02-9.52
Externalizing sample	1.00 (0.10)	0.01-7.66	1.00 (0.26)	0.62-3.37	1.00 (0.29)	0.02-9.52

IPTW, inverse probability of treatment weights; IPCW, inverse probability of censoring weights; SD, standard deviation.
