# Supplementary material for: Impact of prenatal exposure to benzodiazepines and z-hypnotics on behavioral problems at 5 years of age: A study from the Norwegian Mother and Child Cohort Study
Source: PLoS One. 2019 Jun 6;14(6):e0217830. doi: 10.1371/journal.pone.0217830 (PMC6553737; doi:10.1371/journal.pone.0217830)
Supplement: S4 Table — (PDF) [file pone.0217830.s004.pdf]

S4 Table. Balance between exposed and unexposed in the stabilized weighted samples with complete information on the child's internalizing and externalizing behaviors.

**Characteristic**	**Internalizing weighted sample**			**Externalizing weighted sample**		
	Mean/ proportion exposed	Mean/ proportion unexposed	Standardized mean/proportion differences	Mean/ proportion exposed	Mean/ proportion unexposed	Standardized mean/proportion differences
Age, years	30.596	30.181	0.097	30.642	30.177	0.109
Primiparous	0.436	0.456	-0.036	0.433	0.457	-0.043
Married/cohabiting	0.939	0.964	-0.026	0.939	0.964	-0.027
College/university education	0.704	0.655	0.072	0.707	0.656	0.074
Pre-pregnancy BMI, kg/m^2	24.579	24.051	0.114	24.601	24.056	0.118
Smoking	0.045	0.068	-0.024	0.045	0.068	-0.024
Alcohol intake during						

pregnancy						
No or minimal	0.685	0.751	-0.091	0.702	0.751	-0.067
Low to moderate	0.191	0.164	0.032	0.172	0.164	0.010
Frequent	0.124	0.085	0.020	0.126	0.085	0.021
Illicit drug use	0.018	0.007	0.010	0.019	0.007	0.010
Folic acid supplementation	0.659	0.610	0.076	0.669	0.610	0.091
Chronic disease	0.164	0.103	0.069	0.167	0.103	0.072
LTH of MD	0.058	0.061	-0.003	0.057	0.061	-0.004
SCL-5	0.037	-0.002	0.042	0.048	-0.002	0.054
Sleep problems	0.236	0.161	0.093	0.223	0.162	0.075
Mental health problems	0.131	0.116	0.018	0.137	0.116	0.024
Adverse life event						
No	0.407	0.398	0.022	0.409	0.397	0.029
At least one, not painful	0.217	0.246	-0.037	0.227	0.246	-0.025

At least one, painful/very painful	0.364	0.357	0.004	0.364	0.357	0.004
Co-medications during pregnancy						
NSAIDs	0.066	0.064	0.003	0.058	0.064	-0.006
Opioids	0.037	0.021	0.015	0.036	0.021	0.014
Paracetamol	0.499	0.469	0.058	0.494	0.470	0.048
Antidepressants	0.028	0.011	0.016	0.028	0.012	0.016
Antipsychotics	0.014	0.009	0.005	0.014	0.009	0.005
Antiepileptics	0.010	0.003	0.007	0.011	0.004	0.007
Triptans	0.013	0.010	0.003	0.011	0.010	0.001
Boy	0.510	0.512	-0.004	0.527	0.512	0.029

BMI, body mass index; LTH of MD, Life Time History of Major Depression; SCL-5, the Hopkins Symptoms Checklist-5; NSAIDs, nonsteroidal anti-inflammatory drugs.
