# Supplementary material for: Impact of prenatal exposure to benzodiazepines and z-hypnotics on behavioral problems at 5 years of age: A study from the Norwegian Mother and Child Cohort Study
Source: PLoS One. 2019 Jun 6;14(6):e0217830. doi: 10.1371/journal.pone.0217830 (PMC6553737; doi:10.1371/journal.pone.0217830)
Supplement: S5 Table — (PDF) [file pone.0217830.s005.pdf]

S5 Table. Characteristics of mothers with mental health and/or sleep problems, and mothers that used BZD and/or z-hypnotics during pregnancy.

**Characteristics**	**Mental health and/or sleep problems (N=8475)**		**BZDs and/or z-hypnotics users (N=366)**	
Maternal characteristics	**Exposed (N=186)**	**Unexposed (N=8289)**	**Exposed during pregnancy (N=175)**	**Exposed prior to pregnancy only (N=191)**
Age, years; mean ± SD	31.6 ± 4.4	30.7 ± 4.6	31.6 ± 4.3	30.8 ± 4.9
Primiparous, n (% of N)	93 (50.0)	4216 (50.9)	73 (41.7)	104 (54.5)
Married/cohabiting, n (% of N)	168 (90.3)	7892 (95.2)	164 (93.7)	176 (92.1)
College/university education[a], n (% of N)	140 (75.3)	5736 (69.2)	138 (78.9)	124 (64.9)
Pre-pregnancy BMI, $kg/m^{2;}$ mean ± SD	24.1 ± 4.4	24.0 ± 4.2	24.0 ± 4.2	23.5 ± 4.1
Smoking, n (% of N)	25 (13.4)	467 (5.6)	14 (8.0)	21 (11.0)
Alcohol intake during pregnancy[b], n (% of N)				
No or minimal	128 (68.8)	6277 (75.7)	121 (69.1)	140 (73.3)
Low to moderate	43 (23.1)	1352 (16.3)	36 (20.6)	29 (15.2)
Frequent	15 (8.1)	660 (8.0)	18 (10.3)	22 (11.5)
Illicit drug use[c], n (% of N)	7 (3.8)	87 (1.0)	4 (2.3)	9 (4.7)
Folic acid supplementation[d], n (% of N)	118 (63.4)	5478 (66.1)	106 (60.6)	142 (74.3)

Chronic disease[e], n (% of N)	41 (22.0)	1046 (12.6)	32 (18.3)	24 (12.6)
LTH of MD, n (% of N)	46 (24.7)	1216 (14.7)	29 (16.6)	42 (22.0)
SCL-5 [f], mean ± SD	1.2 ± 1.6	0.45 ± 1.2	0.7 ± 1.4	0.7 ± 1.3
Sleep problems, n (% of N)	127 (68.3)	5768 (69.6)	86 (49.1)	62 (32.5)
Mental health problems, n (% of N)	131 (70.4)	3634 (43.8)	78 (44.6)	96 (50.3)
Adverse life event, n (% of N)				
No	31 (16.7)	2431 (29.3)	46 (26.3)	51 (26.7)
At least one, not painful	34 (18.3)	2109 (25.5)	34 (19.4)	36 (18.8)
At least one, painful/very painful	121 (65.1)	3749 (45.2)	95 (54.3)	104 (54.5)
Co-medications during pregnancy, n (% of N)				
NSAIDs	30 (16.1)	674 (8.1)	25 (14.3)	15 (7.9)
Opioids	24 (12.9)	225 (2.7)	21 (12.0)	8 (4.2)
Paracetamol	126 (67.7)	4382 (52.9)	123 (70.3)	112 (58.6)
Antidepressants	55 (29.6)	314 (3.8)	29 (16.6)	16 (8.4)
Antipsychotics	17 (9.1)	100 (1.2)	13 (7.4)	7 (3.7)
Antiepileptics	5 (2.7)	39 (0.5)	4 (2.3)	3 (1.6)
Triptans	9 (4.8)	106 (1.3)	5 (2.9)	5 (2.6)
Child characteristics				
Boy, n (% of N)	96 (51.6)	4201 (50.7)	93 (53.1)	88 (46.1)
Congenital malformation [g], n (% of N)	10 (5.4)	421 (5.1)	8 (4.6)	11 (5.8)
Preterm (<37 weeks) [g], n (% of N)	9 (4.8)	402 (4.8)	14 (8.0)	6 (3.1)
Missing	2 (1.1)	26 (0.3)	2 (1.1)	3 (1.6)
Low birth weight (<2500g)[g], n (% of N)	7 (3.8)	215 (2.6)	13 (7.4)	2 (1.0)

Missing	1 (0.5)	4 (0.05)	1 (0.6)	1 (0.5)

BZDs, benzodiazepines; SD, standard deviation; BMI, body mass index; NSAIDs, nonsteroidal anti-inflammatory drugs; SCL-5, the Hopkins Symptoms Checklist-5; LTH of MD, Life Time History of Major Depression.

[a] Highest level of either completed or ongoing education.

[b] No or minimal alcohol intake (less than once per month); Low to moderate alcohol intake (once per month to once per week); Frequent alcohol intake (more than once per week).

[c] Illicit drug use during pregnancy or the last month before pregnancy; illicit drugs included hash (exposed; unexposed: 3.7%; 0.5%), amphetamine (1.1%; 0.08%), ecstasy (1.1%; 0.02%), cocaine (1.8%; 0.07%) or heroin (0; 0.02%).

[d] Folic acid supplementation in the four weeks before pregnancy or up to week 12 during pregnancy.

[e] Chronic diseases included asthma, diabetes treated with insulin, Crohn’s disease, arthritis, lupus, epilepsy, multiple sclerosis, and cancer.

[f] Presence of depressive or anxiety symptoms, indicated on the 5-item short version of the Hopkins Symptoms Checklist (SCL-5) during gestational week 17 and/or 30.

[g] Not included in the analysis.
