# Supplementary material for: Impact of prenatal exposure to benzodiazepines and z-hypnotics on behavioral problems at 5 years of age: A study from the Norwegian Mother and Child Cohort Study
Source: PLoS One. 2019 Jun 6;14(6):e0217830. doi: 10.1371/journal.pone.0217830 (PMC6553737; doi:10.1371/journal.pone.0217830)
Supplement: S1 Appendix — (DOCX) [file pone.0217830.s006.docx]

**S1 Appendix.**

**Additional details on “Materials and methods”**

Instrument documentation is available at <https://www.fhi.no/en/studies/moba/for-forskere-artikler/questionnaires-from-moba/>.

**Severity of depression and anxiety symptoms**

The Hopkins Symptoms Checklist-25 (SCL-25) is a psychometric scale designed to measure symptoms of anxiety and depression in population surveys [1]. SCL-5 is highly correlated to the SCL-25 [2, 3]. The SCL-5 composes of the following items: “Nervousness or shakiness inside”, “Feeling fearful”, “Feeling hopeless about the future”, “Feeling blue” and “Worrying too much about things”. Women could indicate whether they had been bothered by any of the listed symptoms during the last two weeks in a four category response, from "not bothered" to "very bothered", which are rated 1 to 4, respectively.

**Adverse life events**

Adverse life events from six months prior to pregnancy to third trimester were measured in Q3. Women were asked to indicate whether they had experienced different types of problems (e.g., at work or study place, financial, divorce) or events (e.g., a serious injury or accident, illness, abuse or loss of someone close) during the last 12 months, and how painful/difficult these problems and events were (not too bad; painful/difficult; very painful/difficult). Women were grouped as having experienced none, at least one not painful adverse life event, at least one painful or very painful event. The questions on the MoBa questionnaire were selected primarily because of their relevance to the population in general, partly due to their relevance to women with small children

**The Child Behavior CheckList (CBCL)**

The CBCL is a widely used method of identifying problem behavior in children. The CBCL has adequate sensitivity (71%) and specificity (92%) [4]. The predicative validity has been demonstrated both in Danish and Norwegian samples [5, 6]. The items comprising the scale and reliability coefficients are presented in S5 Table.

**S5 Table. The CBCL items in each sub-scale in MoBa Q-5years and sub-scale reliability (Chronbach’s α).**

|  | **Items** | **Cronbach’s α** |
| --- | --- | --- |
| **Externalizing behavior** |  |  |
| Aggressive behavior | - Can’t stand waiting, wants everything now - Demands must be met immediately - Doesn’t seem to feel guilty after misbehaving - Defiant - Gets in many fights - Hits others - Punishment doesn’t change his/her behavior | 0.71 |
| Attention problems | - Can’t concentrate, can’t pay attention for long - Quickly shifts from one activity to another - Can’t sit still, restless or overactive - Poorly coordinated or clumsy | 0.61 |
| **Internalizing behavior** |  |  |
| Anxious/depressed | - Too fearful or anxious - Clings to adult or too dependent - Gets too upset when separated from parents - Nervous, high strung or tense - Unhappy, sad or depressed | 0.62 |
| Somatic complaints | - Does not eat well - Stomach aches or cramps (without medical cause) - Vomiting, throwing up (without medical cause) | 0.22 |
| Emotionally reactive | - Disturbed by any change in routine | - |

**References**

1. Strand BH, Dalgard OS, Tambs K, Rognerud M. Measuring the mental health status of the Norwegian population: A comparison of the instruments SCL-25, SCL-10, SCL-5 and MHI-5 (SF-36). Nord J Psychiatry. 2003;57(2):113-8. doi: 10.1080/08039480310000932.

2. Tambs K, Moum T. How well can a few questionnaire items indicate anxiety and depression? Acta Psychiatr Scand. 1993;87(5):364-7. doi: doi:10.1111/j.1600-0447.1993.tb03388.x.

3. Fink P, Ørnbøl E, Huyse FJ, de Jonge P, Lobo A, Herzog T, et al. A brief diagnostic screening instrument for mental disturbances in general medical wards. J Psychosom Res. 2004;57(1):17-24.

4. Achenbach TM. Manual for the Child Behaviour Checklist/2-3 and 1992 Profile. Burlington, VT: University of Vermont Department of Psychiatry, 1992.

5. Nøvik TS. Validity of the child behaviour checklist in a Norwegian sample. Eur Child Adolesc Psychiatry. 1999;8(4):247-54. doi: 10.1007/s007870050098.

6. Bilenberg N. The Child Behavior Checklist (CBCL) and related material: standardization and validation in Danish population based and clinically based samples. Acta Psychiatr Scand. 1999;100(S398):2-52. doi: 10.1111/j.1600-0447.1999.tb10703.x.
