# Supplementary material for: Impact of prenatal exposure to benzodiazepines and z-hypnotics on behavioral problems at 5 years of age: A study from the Norwegian Mother and Child Cohort Study
Source: PLoS One. 2019 Jun 6;14(6):e0217830. doi: 10.1371/journal.pone.0217830 (PMC6553737; doi:10.1371/journal.pone.0217830)
Supplement: S2 Appendix — (DOCX) [file pone.0217830.s007.docx]

**S2 Appendix.**

**Bias analysis.**

The observed crude OR was 1.79, 95% CI: 1.29-2.48. The selection proportions in the 5-year sample based on the prevalence of externalizing problems at 3 years in the 3-year sample are shown in S6 Table. These proportions yielded a selection OR of 1.05 and a selection‑bias‑corrected OR of 1.70.

S6 Table. Selection proportions in the 5-year sample based on the prevalence of externalizing problems at 3 years in the 3-year sample.

| Exposure to BZD and/or z-hypnotic | Externalizing problems at 3 years | No externalizing problems at 3 years |
| --- | --- | --- |
| Exposed | 0.53 | 0.54 |
| Unexposed | 0.56 | 0.60 |

BZD, benzodiazepine.

S1 Fig shows how the selection OR, and thus, the selection-bias-corrected OR, varied with lower and higher selection proportions of children with externalizing problems that were exposed to BZDs and/or z-hypnotics prenatally. The other selection proportions remained constant, at the levels based on the prevalence of externalizing problems at 3 years, shown in S6 Table.

S2 Fig shows how the selection OR, and thus, the selection-bias-corrected OR, varied with lower and higher selection proportions of children with externalizing problems that were not exposed to BZDs and/or z-hypnotics prenatally. The other selection proportions remained constant, at the levels based on the prevalence of externalizing problems at 3 years, shown in S6 Table.

For the probabilistic analysis, the following selection proportions were used: min OR: 0.74, mode1 OR: 1.02, mode2 OR: 1.25, max OR: 1.59. These proportions resulted in a corrected OR: of 1.56, 95% CI: 1.20-2.15 (conventional OR: 1.79, 95% CI: 1.29-2.48).
