# Supplementary figures and images for: Impact of prenatal exposure to benzodiazepines and z-hypnotics on behavioral problems at 5 years of age: A study from the Norwegian Mother and Child Cohort Study
Source: PLoS One. 2019 Jun 6;14(6):e0217830. doi: 10.1371/journal.pone.0217830 (PMC6553737; doi:10.1371/journal.pone.0217830)

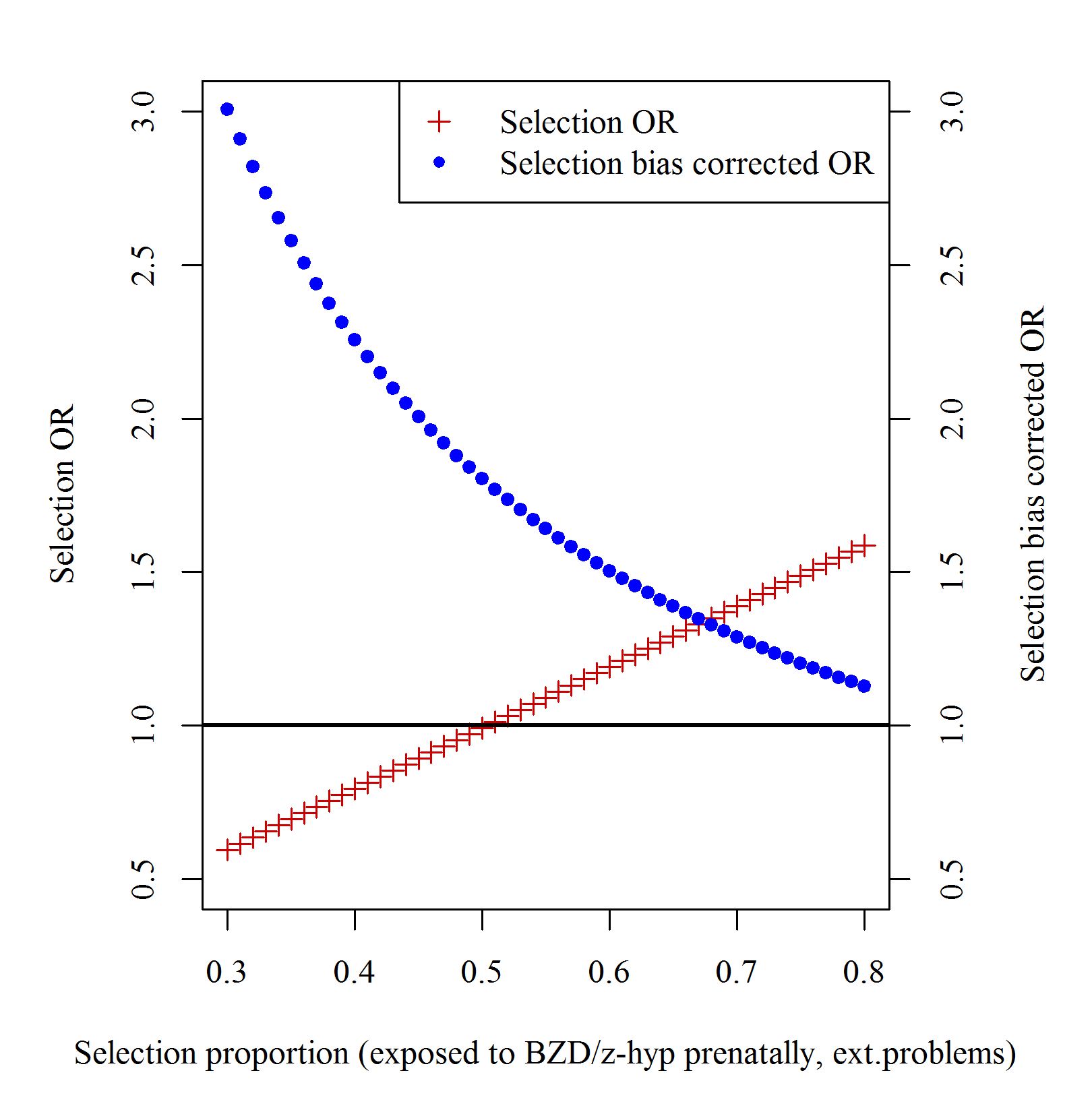

Supplement: S1 Fig — BZD, benzodiazepine; z-hyp, z-hypnotics; Ext. problems, externalizing problems; OR, odds ratio. (TIF) [file pone.0217830.s008.tif]

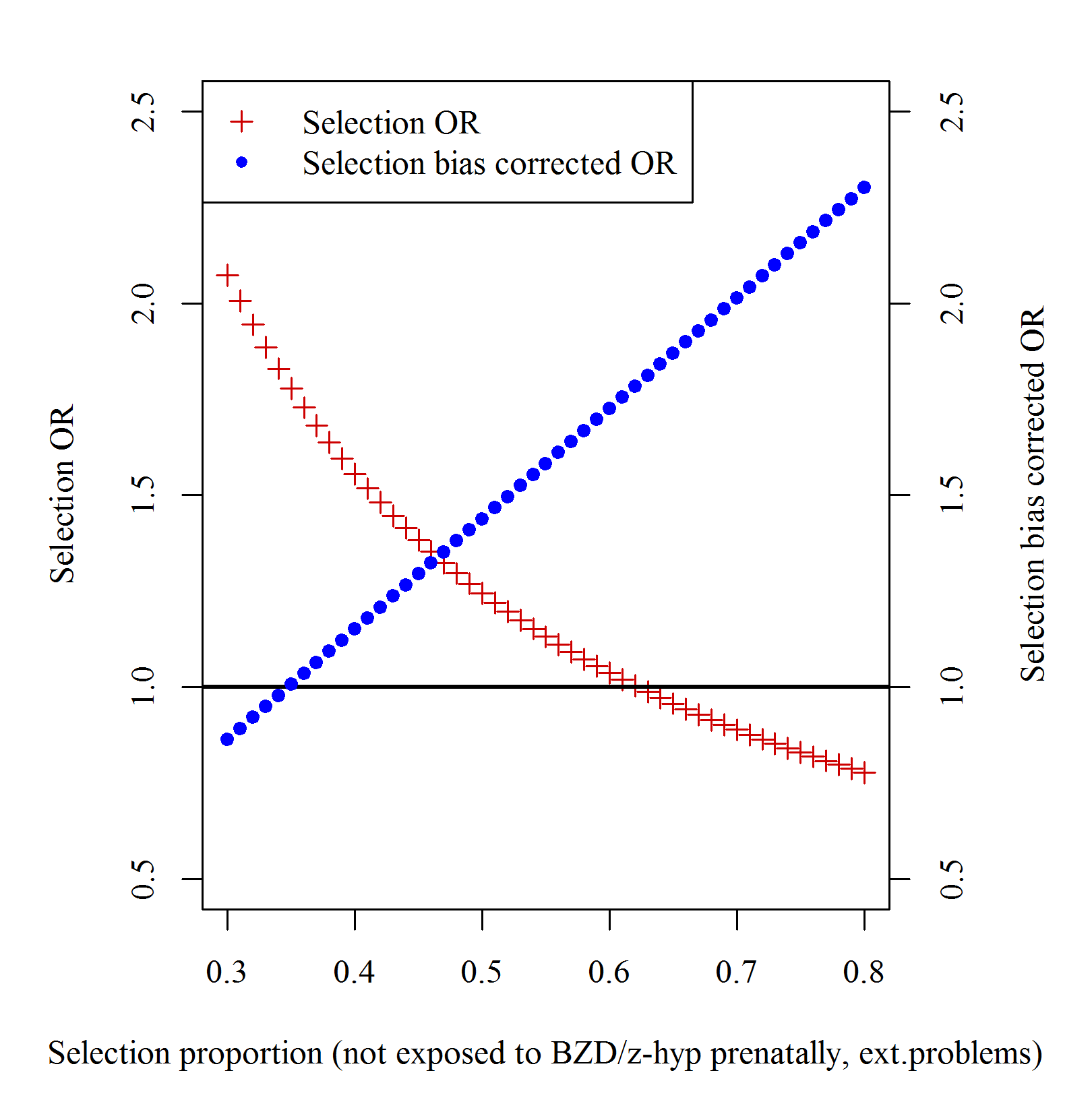

Supplement: S2 Fig — BZD, benzodiazepine; z-hyp, z-hypnotics; Ext. problems, externalizing problems; OR, odds ratio. (TIF) [file pone.0217830.s009.tif]
